# The Measurement of Positive Valence Forms of Empathy and Their Relation to Anhedonia and Other Depressive Symptomatology

**DOI:** 10.3389/fpsyg.2019.00815

**Published:** 2019-04-12

**Authors:** Sharee N. Light, Zachary D. Moran, Carolyn Zahn-Waxler, Richard J. Davidson

**Affiliations:** ^1^Positive Affective Neuroscience Laboratory, Department of Psychology, Georgia State University, Atlanta, GA, United States; ^2^Mendota Mental Health Institute, Madison, WI, United States; ^3^Center for Healthy Minds, University of Wisconsin–Madison, Madison, WI, United States

**Keywords:** positive-valence empathy, anhedonia, hedonic capacity, Beck Depression Inventory-II, empathic concern

## Abstract

Construct validity of a brief self-report measure of “positive-valence empathy” (the tendency to exude positive emotion as a means to stimulate positive affect in others, and/or to vicariously share in another’s positive emotion; Light et al., 2009) was attained utilizing a sample of 282 healthy adults. Positive-valence empathy may have unique predictive ability for differentiating depression versus depression with anhedonia. Confirmatory factor analyses revealed a two-factor structure for the final 15-item Light-Moran Positive Empathy Scale (PES), with an 8-item “Empathic Happiness” subscale (e.g., “I find that other people’s happiness easily rubs off on me”) and a 7-item “Empathic Cheerfulness” subscale (e.g., “I enjoy making others feel good”). “Empathic Happiness” was a significantly better predictor of overall depressive symptomatology (Beck et al., 1996) than anhedonia (Snaith et al., 1995). The Light-Moran PES-15 may have real-world impact and predictive utility for well-being.

## Introduction

Anhedonia – the reduced ability to experience positive emotions – is a key feature of Major Depressive Disorder (MDD) (Heller et al., 2009, 2012; Light et al., 2011). To date, the relative lack of attention to the varieties of positive affect and how they may be impaired in MDD may in part be responsible for the difficulty in developing treatments that are universally effective at targeting MDD and other disorders in which positive affect deficits are prominent. Although anhedonia is one of two possible primary diagnostic criteria (the other is sad mood) that must be present for the diagnosis of MDD to be made, most of the pharmacological treatments currently available do not address this symptom; failing in approximately 30% of patients with MDD (Rush et al., 2006).

Anhedonia is a significant problem, yet we do not have sufficient means to measure all of the facets of this symptom (Ho and Sommers, 2013). The lifetime prevalence of anhedonia in MDD is 5.2% (First et al., 1990), and recent reports estimate that approximately 37% of individuals diagnosed with MDD experience clinically significant anhedonia (Pelizza and Ferrari, 2009). We are only beginning to understand the nuances of anhedonia. The DSM-V states that individuals meeting criteria for anhedonia may report feeling “less interested in hobbies, ‘not caring anymore,’ or not feeling any enjoyment in activities that were previously considered pleasurable,” and “family members often notice social withdrawal or neglect of pleasurable avocations” (American Psychiatric Association [APA], 2013) (p. 163).

Anhedonia is important to recognize and diagnose because it has troubling prognostic value (Spijker et al., 2001), with most research suggesting that anhedonia persists beyond resolution of negative affect in depression – and importantly for our overarching aim to increase our ability to match individuals to targeted treatments based on an accurate assessment of their particular symptom profile – is the fact that anhedonia is a significant prodromal symptom, and a predictor of relapse in adult (Iacoviello et al., 2010) and adolescent samples (McMakin et al., 2013; Rubin, 2013).

The present work specifically addresses “positive-valence empathy” (i.e., the tendency to exude positive emotion as a means to stimulate positive emotion in another person, and/or to share in another’s positive emotion) (Light et al., 2009) as a novel and useful construct to measure as an adjunct to measures that tap anhedonia proper. We focus on “positive-valence empathy” to facilitate the development of better behavioral and pharmacological treatments for anhedonic patients, with the present research project designed to formally assess the putative link between positive-valence empathy and anhedonia in a healthy sample with intent to apply it to clinical samples in the future. Specifically, we hypothesize that positive-valence empathy may have treatment potential; i.e., when evoked, positive-valence empathy may actually antagonize the experience of anhedonia.

Some may question the use of a healthy sample in the present work. However, examining anhedonia and other depressive symptoms that do not reach diagnostic significance represents a more stringent test of our hypotheses about the relationship between positive-valence empathy and anhedonia given that any significant effects would be more difficult to achieve given the fairly restricted range and variability inherent in a non-clinical sample. More importantly, however, given that anhedonia represents a putative endophenotype (i.e., a trait that is associated with the expression of an illness and represents the genetic liability of a disorder in non-affected individuals), there is reason to suggest that even healthy individuals vary in their level of anhedonia along a continuum (Harvey et al., 2007). Overall then, this study is best thought of as capturing “everyday” anhedonia symptoms as they occur in the general population (Harvey et al., 2007).

The National Institute of Mental Health’s (NIMH) Research Domain Criteria (RDoC) has provided a compelling framework for conceptualizing symptomatology such as anhedonia that cuts across DSM diagnoses (Cuthbert, 2014). Here we focus in on the RDoC “Positive Valence Systems” aspect of RDoC because we believe that, as mentioned previously, anhedonia is a viable candidate for an underlying endophenotype for neuropsychiatric dysfunction, as it appears across several psychiatric and neurological disorders (e.g., MDD, Parkinson’s disease, schizophrenia, dementia, and TBI). Thus, anhedonia is transdiagnostic though it is perhaps a cardinal symptom of MDD, and therefore its relationship to depression was chosen as the focus here.

To facilitate the empirical measurement of positive-valence empathy in relation to anhedonia, the development of an accurate, simple to administer, and psychometrically sound means for detecting *variability* in positive-valence empathy at pre-treatment may be a very important strategy for characterizing the need for treatment, and tracking an individual’s treatment response, and may ultimately spur the development of new behavioral treatments and/or antidepressants; if this construct proves to be mutable pre- to post-treatment. Therefore, our overarching aim was to provide initial validation of the construct of positive-valence empathy in a healthy sample. A self-report measure of the construct should show:

(1) Convergent validity with a well-validated hedonic capacity measure, given the putative relationship between one’s ability to experience joy first hand/first-person (i.e., first-person joy/happiness refers to an individual’s ability to enjoy a rewarding stimulus primarily directed toward oneself, or joy experienced individually; for example, the enjoyment derived from reading a good book, eating a delicious meal, watching a sunset, or watching a pleasant video clip) as a potential correlate for the ability to relish in the joy of someone else. For example, a deficit in positive empathy may or may not also signal a deficit in first-person joy/happiness, and having an understanding of the possibility of such a dissociation could be useful clinically; i.e., a patient may need to focus on making gains in the experience of first-person joy *and* positive empathy (e.g., the patient may struggle with deriving pleasure from basic self-focused rewards and empathic situations), or may only need to make gains in positive empathy skills (if their basic first-person positive emotional skills are relatively intact). Therefore, it would be important to measure both aspects of hedonic responsivity (i.e., first-person hedonic capacity and positive empathic hedonic capacity) in order to better characterize the patient’s individual treatment needs.
(2) Convergent validity with other empathy measures (e.g., that tap empathic concern – the tendency to experience sadness or tender feelings for others who are suffering).
(3) The existence of two distinct, yet positively correlated, *subtypes* of positive-valence empathy (described in detail in the next section) – empathic cheerfulness and empathic happiness – using principal components analysis (PCA).
(4) Discriminant validity; the construct of positive-valence empathy should be distinct from depression proper.

### Two Subtypes of Positive-Valence Empathy

An individual may express positive emotion while in the presence of someone who is experiencing a negative emotional state as a means to alleviate the negative emotion that person is feeling by catalyzing a positive emotional state in that person (e.g., the observer tries to “cheer” the target up). Similarly, an individual may express positive emotion as a means to induce a state of joy in another person who is in a neutral or content emotional state for its own sake. This subtype of positive-valence empathy can be referred to as *empathic cheerfulness*. Furthermore, an individual may vicariously experience pleasure in response to someone else’s positive emotion (e.g., an observer feels vicarious joy at a wedding or birthday party). This subtype of positive-valence empathy can be referred to as *empathic happiness*.

### Positive-Valence Empathy and Anhedonia

It is proposed here that positive-valence empathy and anhedonia are antithetical constructs, and in fact, positive-valence empathy may be a useful means by which to work with patients who are anhedonic (i.e., learning to experience positive affect vicariously may be one route toward relieving anhedonia). Positive-valence empathy, as a scientific construct, is based on the idea that humans have the capacity (and perhaps the propensity) to share in the positive affect of other people, and the intact presence of this ability may be protective psychologically. Furthermore, deficits in this ability may be reversible, and gains made in positive-valence empathy may contribute to reduction in overall anhedonia. As the parsing of emotional processes becomes ever more refined, an investigation of the processes by which positive affect can be transmitted between people (i.e., how positive affect gets under the skin) could prove to be a useful endeavor for the purposes of developing *treatments* for various mood disorders – particularly Major Depressive Disorder and Persistent Depressive Disorder (i.e., dysthymia) – and other conditions that affect a person’s basic interest in life and/or their subjective experience of positive emotional states.

### The Light-Moran Positive Empathy Scale (PES)

The present work was designed to create a paper-and-pencil analog for the positive-valence empathy construct which can be used in conjunction with functional magnetic resonance imaging in the future to interrogate the neural correlates of positive empathy in healthy and clinical samples. Though previous measures have been established for the quantification of empathic concern, such as the “Interpersonal Reactivity Index (IRI)” (Davis, 1996) and the “Empathy Quotient (Baron-Cohen and Wheelwright, 2004; Lawrence et al., 2004)” (but note: this measure contains items pertinent to social aptitude, perspective-taking, and empathic concern), we sought to develop a brief self-report measure of *positive-valence empathy*. The scale developed differs from the “Empathy Quotient” and the “Interpersonal Reactivity Index” because all of the items relate to positive emotional responses to others emotional displays, and we only attempt to measure the *emotional* component of empathy whereas the EQ and IRI measure the cognitive and emotional components of empathy.

When developing the original (pre-factor analysis) 41-items for the scale, we hypothesized that positive-valence empathy would be observable via two underlying behaviors: (1) *empathic happiness*, or cases in which someone tends to respond with positive affect, evincing pleasure, in response to another’s positive experience (e.g., “I feel pleasure in watching other people open gifts”), and (2) *empathic cheerfulness*, or cases in which someone exhibits positive affect as a means to catalyze a positive mood state in another who is dysphoric or neutral (e.g., “I get a lot of pleasure from making other people feel good”).

### Hypotheses

We were particularly interested in investigating the relation between positive-valence empathy and anhedonia given the putative relationship between capacity to experience personal pleasure as an important correlate to any such experience vicariously. We predicted a positive correlation between positive-valence empathy and pleasure capacity as measured by the Snaith-Hamilton Pleasure Scale (Snaith et al., 1995) (i.e., convergent validity).

As an additional validity measurement (i.e., discriminant validity), we chose to investigate the relation between negative affect and positive-valence empathy by examining the relationship between scores on our measure and scores on the Beck Depression Inventory-II (Beck et al., 1996; Steer et al., 1999) – a scale that assesses overall severity of depressive symptoms.

However, first, we sought to validate our measure of positive-valence empathy via principal component analysis (PCA). We expected the scale to have two factors. Indeed, the *Light-Moran Positive Empathy Scale* (PES) is composed of items designed to tap empathic happiness and empathic cheerfulness. Planned confirmatory factor analyses utilizing PCA extraction and oblique rotation were performed. Then the scale was examined in relation to other constructs of interest, namely hedonic capacity/anhedonia and depressive symptomatology.

In sum, a consideration of a wider variety of positive affective states, e.g., empathic happiness, and empathic cheerfulness – beyond the study of “happiness” *per se* – is warranted. Our hypotheses center on the idea that positive-valence empathy should relate to anhedonia, and individuals who are anhedonic and/or demonstrate heightened depressed symptoms will score lower on both aspects of positive-valence empathy.

## Materials and Methods

### Participants

Two-hundred and twenty-six participants responded to either email advertisements or flyers posted throughout the general University of Wisconsin–Madison area in 2007–2008. In either scenario, individuals completed an online survey in exchange for being entered into a raffle for a chance to win a free digital music player. We retained data from a total of 214 participants after excluding 12 people who aborted the survey without answering all items. Of these, 67 were male (31.3%), 147 were female, and all were aged between 18 and 56 years (*M* = 22.45, *SD* = 6.25). The majority of these participants were students (80.89%). The sample consisted of individuals of Caucasian (84.44%), Asian (9.78%), Hispanic/Latino (1.78%), and African (1.33%) descent, with 2.67% of our participants reporting a mixed racial heritage.

An additional 68 participants contributed data in 2011–2012. Of these, 23 were male (34%), and all were age 18–63 (*M* = 25.68, *SD* = 10.64). Half of our participants were undergraduate students (51.47%). 20.59% of participants were college graduates, 17.65% were high school graduates or had obtained their GED, and 10.30% had obtained a graduate degree (e.g., masters, PhD, MD, JD, etc.). Around 74% of participants were white, 8.82% of participants were Asian, 8.82% of participants were African-American, 7.4% of participants were Hispanic, and 1.4% of participants were of Native American descent.

In total, 282 adults contributed data. All participants provided informed consent (written) and all aspects of this study were approved by the University of Wisconsin–Madison Institutional Review Board (IRB) and were in compliance with the Declaration of Helsinki.

### Measures

#### Positive-Valence Empathy

The *Light-Moran PES* utilizes a Likert scale, i.e., extremely untrue (=1), quite untrue (=2), slightly untrue (=3), neither true nor false (=4), slightly true (=5), quite true (=6), extremely true (=7). Item examples include: “I very much enjoy and feel uplifted by happy endings” (i.e., Empathic Happiness) and “I enjoy helping people to see that they can turn “lemons into lemonade” (i.e., Empathic Cheerfulness). The total score on the PES is calculated by summing all items. Subscale scores were computed by summing items 1, 3, 5, 6, 7, 8, 14, and 15 (Empathic Happiness); or summing items 2, 4, 9, 10, 11, 12, and 13 (Empathic Cheerfulness) (see Appendixes A,B). Higher scores indicate greater empathic ability.

The original items for the scale were generated based on the following criteria: A panel composed of two researchers independently rated – on a Likert scale from 0 to 2, with 2 representing full agreement, 1 representing partial agreement, and 0 representing a lack of agreement – each of the following: (1) is this item tapping an essential feature of the construct? (2) is it useful, but not essential?, or (3) is it not necessary in assessing the relevant trait? Items that were not agreed upon (i.e., scored a “0”) were eliminated.

#### Validation

Participants also completed the *Empathy Quotient* (EQ) (Baron-Cohen and Wheelwright, 2004; Lawrence et al., 2004), which constituted the primary means by which construct validity of the PES was assessed. The EQ is composed of 28-items. Each participant had to indicate their agreement with statements pertaining to their general tendency toward social interaction, perspective taking, and empathic concern. An example of an item on the EQ is: “I get upset if I see people suffering on news programs.” Higher scores indicate greater empathic ability.

As an additional validation check, a subset of the sample (68 participants) also completed the “Empathic Concern” subscale from the *Interpersonal Reactivity Index* (Davis, 1996). Higher scores on this measure indicate greater empathic concern.

#### Social Desirability

The Marlowe-Crowne Social Desirability Scale (SDS) (Crowne and Marlowe, 1960) is a self-report measure designed to quantify the tendency of individuals to project a favorable image of themselves during social interaction. The scale contains 33 true-false items that describe both acceptable but improbable behaviors, as well as unacceptable but probable behaviors. Higher scores indicate a greater propensity for responding in a socially desirable manner.

#### Hedonic Capacity

The Snaith-Hamilton Pleasure Scale (SHAPS) (Snaith et al., 1995) is a 14-item scale used to measure levels of anhedonia present over the “last few days” and is listed as a measure of “sustained/longer-term responsiveness to reward attainment” by the RDoC website. Participants choose one of four responses for each item, i.e., Definitely Agree (=4), Agree (=3), Disagree (=2), and Definitely Disagree (=1). Higher scores reflect greater pleasure capacity (i.e., lower anhedonia). Originally, the authors of the scale recommended a scoring system whereby the four response categories are recoded dichotomously into agree (=0) or disagree (=1). However, following the advice of Franken et al. (2007), we opted to calculate a total score by using the above mentioned 4-point scale for each item. In doing so, we allowed for a greater dispersion of the data given the relatively few number of items. Item examples include: “I would enjoy a cup of tea or coffee or my favorite drink” and “I would find pleasure in small things, e.g., a bright sunny day, a telephone call from a friend.” Scores can range from 14 to 56.

#### Depressive Symptoms

The Beck Depression Inventory-II (BDI-II) (Beck et al., 1996) is a 21 item multiple choice self-report measure that is based on Beck’s “triad of negative cognitions” pertaining to the world, the future, and the self. Thus, the development of the BDI-II reflects that in its structure, with items such as “I have lost all of my interest in other people” to reflect the world, “I feel discouraged about the future” to reflect the future, and “I blame myself for everything bad that happens” to reflect the self. The structure of the BDI-II is also based on the idea that depression is composed of two components: an affective component (e.g., mood) and a physical or “somatic” component (e.g., loss of appetite). The items that pertain to the affective component include the following eight items: pessimism, past failures, guilty feelings, punishment feelings, self-dislike, self-criticalness, suicidal thoughts or wishes, and worthlessness. The items that pertain to the somatic component are the remaining thirteen items, including: sadness, loss of pleasure, crying, agitation, loss of interest, indecisiveness, loss of energy, change in sleep patterns, irritability, change in appetite, concentration difficulties, tiredness and/or fatigue, and loss of interest in sex.

We were primarily interested in the BDI to assess *severity* of symptoms rather than particular symptom clusters. Indeed, recent research suggests that the BDI-II cannot be reliably broken into subdomains (McElroy et al., 2018); and these and other researchers (e.g., Reise et al., 2010, 2013) recommend using the total score in research and clinical practice. Higher scores indicate more severe depression; a total score of 0–13 is considered minimal, 14–19 is mild, 20–28 is moderate, and 29–63 is severe.

### Procedure

The 226 participants recruited in 2007–2008 completed the scales online. Upon beginning the survey, all respondents electronically gave their informed consent for participation. No participant was granted access to the remainder of the survey unless s/he did so. Average response time was approximately 30 min. An additional 68 participants completed the 15-item Light-Moran PES (amongst other measures) via computer in the laboratory as part of a comprehensive study not reported on here. All respondents gave their written informed consent for participation.

#### Factor Analysis

Principal components analysis yields one or more composite variables that capture much of the information originally contained in a larger set of items. The components are weighted sums of the original items. Components account for a portion of the total variance among the original variables. Oblique rotation is useful when the underlying latent variables are believed to correlate somewhat with each other.

## Results

### Cohort Effects

An ANOVA was run to investigate cohort effects. Gender, and both positive-valence empathy subtypes, i.e., empathic happiness and empathic cheerfulness, did not differ statistically between the two cohorts (all *p*’s > 0.852). However, the groups did differ in terms of SHAPS score and age (but not gender) such that the larger group of 214 was more anhedonic, and the smaller group of 68 was significantly older. The minimum SHAPS score in the larger sample was 27, whereas the minimum score in the smaller sample was 30. This adds important variability to the data set; thus, we elected to keep the groups combined in analyses to maximize generalizability to a broader swathe of the hedonic capacity continuum.

**FIGURE 1:**
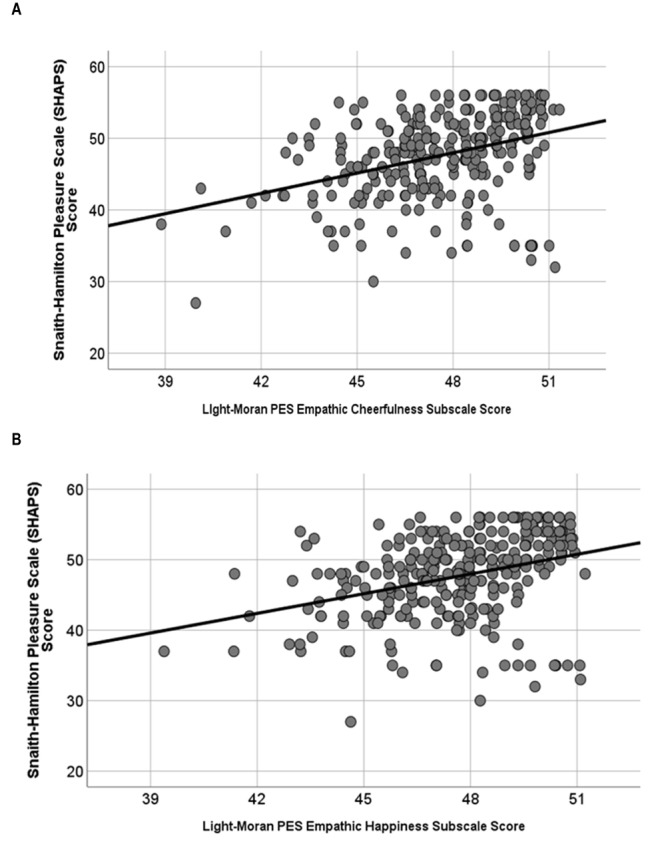
**(A)** Empathic cheerfulness [*R*^2^ = 15%, *F*(2,279) = 24.28, *p* < 0.001] and **(B)** empathic happiness [*R*^2^ = 12%, *F*(2,279) = 19.59, *p* < 0.001] were about equally predictive of anhedonia score (measured via the SHAPS).

Concerning age, given the significant difference between groups, we ran regression analyses with age as a covariate where appropriate (i.e., Figure 1A,B), and the results and our interpretation remained the same when looking at the groups separately versus combined.

### Positive Empathy Scale – 41-Item Version

#### Data Screening

The mean score on the 41-item PES was 243 (*SD* = 31.71). The distribution was leptokurtic (*Kurtosis statistic* = 1.62, *SE* = 0.33), and negatively skewed (*skewness statistic* = -0.83, *SE* = 0.17).

#### Validity/Reliability

Reliability was very high (Cronbach’s α = 0.96). Initially, we sought to establish factor validity of the PES in terms of a two-factor model, with one factor corresponding to *empathic happiness* and the other factor corresponding to *empathic cheerfulness*. In order to test this hypothesis, we implemented a PCA using oblique rotation and extracted two factors; one accounted for 18.18% and the other accounted for 24.49% of the total variance, for a total of 42.67% of the variance explained. Thus, the analysis supported the existence of a two-factor model, as items that loaded highly on each factor generally discriminated between *empathic happiness* and *empathic cheerfulness*. Next we sought to shorten the questionnaire.

### Light-Moran Positive Empathy Scale – 15-Item Version

In re-reviewing the items, we attempted to reduce the questionnaire based upon three criteria: (1) clarity of expression – i.e., whether the item explicitly and unambiguously expressed the emotions that were being held by both the subject and target of empathy; (2) clarity of the divide between our two *a priori* factors, i.e., which items best capture *empathic happiness* and which items best capture *empathic cheerfulness*?, and (3) items that correlated less than 0.30 with the scale, were eliminated from the scale.

The end result was a shorter PES containing 15 of the original 41-items (see Appendix A for the 15-item Light-Moran PES). The mean score on the 15-item PES was 88 (*SD* = 10.94), with a median of 89. The distribution was leptokurtic (*Kurtosis statistic* = 2.00, *SE* = 0.33), and negatively skewed (*skewness statistic* = -0.98, *SE* = 0.17). When 1 outlier with a PES score of 35 was removed, the mean PES score remained 88 (*SD* = 10.35), the median remained 89; the distribution was more mesokurtic (*Kurtosis statistic* = 0.11, *SE* = 0.33); yet still negatively skewed (*skewness statistic* = -0.61, *SE* = 0.17).

Using all 282 participants, the 15-item PES maintained a very high inter-item reliability (Cronbach’s α = 0.92). Furthermore, the PCA with oblique rotation performed on the 15-item PES once again confirmed a two-factor structure, with excellent discrimination between *empathic happiness* and *empathic cheerfulness*. Cronbach’s alpha for each subscale was as follows: 0.84 for *empathic cheerfulness* and 0.87 for *empathic happiness*. The factor analysis of the 15-item PES was more successful in explaining variance in the underlying construct of interest (56.23%), relative to the 41-item PES. Additionally, each of the 15 items produced significant factor loadings (factor loadings ranged from -0.42 to 0.96; Table 1). Kaiser-Myer-Olkin Measure of Sampling Accuracy was above 0.50 (*KMO* = 0.941), and Bartlett’s Test of Sphericity was significant (*p* < 0.001); confirming sampling adequacy and good fit to the data, respectively.

**Table 1:** Factor matrix.

Factor matrix for 15-item Positive Empathy Scale (PES-15) *N* = 282		
	Factor 1	Factor 2
	(empathic	(empathic
PES Item #	happiness)	cheerfulness)
14 “I easily get excited when those around me are lively and happy”	0.711	0.050
11 “I enjoy making others feel good”	0.096	–0.767
5 “I also feel good when someone I know feels good”	0.60	–0.197
7 “It often makes me feel good to see the people around me smiling”	0.609	–0.268
15 “I can’t help but smile when my friends smile at me”	0.719	–0.043
10 “I feel good when I know I have pleased someone”	–0.133	–0.922
1 “I very much enjoy and feel uplifted by happy endings”	0.462	–0.155
2 “I like to tell people nice things to make them feel good”	0.209	–0.608
4 “I feel great when I find out that I have made someone else happy”	0.026	–0.822
5 “I enjoy hearing about my friends’ good days”	0.670	–0.128
12 I enjoy helping a person change their bad mood into a good mood	0.350	–0.415
9 “I enjoy helping people to see that they can turn ‘lemons into lemonade”’	0.50	–0.223
3 “I can’t stop myself from laughing when others are doing so”	0.759	0.123
13 “I enjoy making others laugh”	0.266	–0.469
8 “I find that other people’s happiness easily rubs off on me”	0.958	0.193

#### Convergent Validity

Higher scores on the Light-Moran PES-15 were associated with higher scores on the *Empathy Quotient*, even with *Social Desirability* score entered as a covariate [*F*(2,212) = 42.95; *R*^2^ = 29%, *p* < 0.001], which suggests that the PES-15 is a valid measure of empathy, and also provides evidence that positive empathy relates positively to general empathy.

In addition, when looking at the data from the 68 participants who completed the *Interpersonal Reactivity Index-Empathic Concern* subscale and the PES-15, empathic *concern* correlated positively with *positive empathy* even with *Social Desirability* entered as a covariate [*F*(2,67) = 9.03; *R*^2^ = 22%, *p* < 0.001].

### Demographic Analyses

#### Gender

A multivariate ANOVA revealed that women (*M* = 89.63, *SD* = 9.74) scored higher than men (*M* = 83.95, *SD* = 10.68) on the PES-15.

#### Ethnicity

A multivariate ANOVA revealed similar scores across ethnicity groups for the PES-15 [*F*(8,416) = 1.4, *p* = 0.18].

#### Age

There was no correlation between age and PES-15 score (*r* = -0.07, *p* = 0.34).

#### Occupation

A multivariate ANOVA revealed similar scores on the PES-15 across participants who were students and participants who were not [*F*(2,210) = 0.09, *p* = 0.92].

### The Relationship Between Empathy and Anhedonia

A greater *general* pleasure capacity (i.e., the ability to enjoy a wide range of positive stimuli; i.e., less anhedonia) – as reflected by a greater Snaith-Hamilton Pleasure Scale score – predicted greater total *positive-valence empathy* across the sample as a whole even with *Social Desirability* entered as a covariate [total sample: *R*^2^ = 13%; *F*(2, 212) = 15.03; *p* < 0.001; Men: *r* = 0.38, *p* < 0.001; Women: *r* = 0.31, *p* < 0.001], and to a much lesser extent, greater general empathy (as measured by the EQ) with *Social Desirability* entered as a covariate [total sample: *R*^2^ = 3%, *F*(2,213) = 3.3; *p* = 0.04].

Both *Empathic Cheerfulness* [*R*^2^ = 15%, *F*(2,279) = 24.28, *p* < 0.001; Figure 1A] and *Empathic Happiness* [*R*^2^ = 12%, *F*(2,279) = 19.59, *p* < 0.001; Figure 1B] were about equally predictive of anhedonia score, even with age entered as a covariate.

### The Relationship Between Empathy and General Depressive Symptoms

**FIGURE 2:**
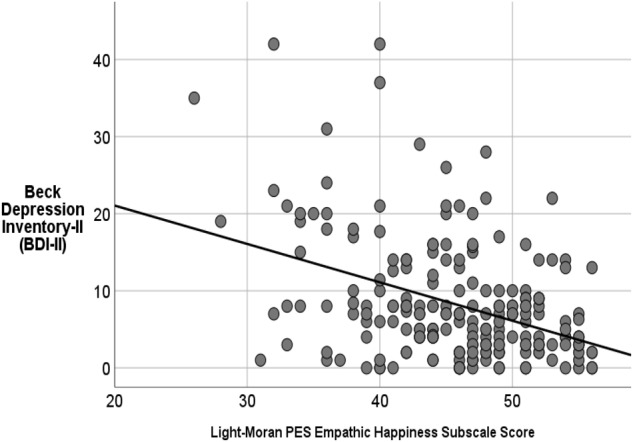
Greater *empathic happiness* score uniquely predicts lower depression (as measured by the BDI-II) better than anhedonia (as measured by the *SHAPS*) or *empathic cheerfulness* even with Social Desirability included in the model as a covariate (*R*^2^ = 16%, *p* < 0.001).

Greater capacity for *positive-valence empathy* relates to lower overall depression (*r* = -0.33, *p* < 0.001, Table 2), as does *empathic concern* as measured by the *Empathy Quotient* (*r* = -0.27, *p* < 0.001). When we looked at the relation between *BDI-II score* and the *positive-valence empathy* subscales – with age included as a covariate – we discovered that *Empathic Happiness* (β = -0.51, *p* < 0.001) was a better predictor of BDI-II score relative to *Empathic Cheerfulness* (β = -0.20, *p* < 0.001). Furthermore, using a stepwise regression, we were able to determine that the *Empathic Happiness* subscale score was also a significantly better predictor of BDI-II score [*R*^2^ = 16%, *F*(2,212) = 19.51; *p* < 0.001; Figure 2] than hedonic capacity/anhedonia as measured by the SHAPS when both predictors were entered into the same regression model, even with *Marlowe-Crowne Social Desirability* score included as a covariate (combined model: *R*^2^ change when SHAPS score added = 0.02, ns).

**Table 2:** Correlations.

	Positive Empathy Scale-PES-15 item total (*N* = 282)	Positive Empathy Scale-PES-15 item Empathic Happiness subscale (*N* = 282)	Positive Empathy Scale-PES-15 item Empathic Cheerfulness subscale (*N* = 282)
Marlowe-Crowne Social Desirability Scale (SDS)	0.20	0.188	0.184
*M* = 14.09 (*SD* = 2.15) Range = 10–20	(*p* < 0.01)^∗∗^	(*p* < 0.01)^∗∗^	(*p* < 0.01)^∗∗^
Empathy Quotient (EQ)	0.53	0.451	0.551
*M* = 32.29 (*SD* = 8.88) Range = 6–53	(*p* < 0.001)^∗∗∗^	(*p* < 0.001)^∗∗∗^	(*p* < 0.001)^∗∗∗^
Snaith-Hamilton Pleasure Scale (SHAPS)	0.35	0.312	0.33
*M* = 46.95 (*SD* = 6.04) Range = 27–56	(*p* < 0.001)^∗∗∗^	(*p* < 0.001)^∗∗∗^	(*p* < 0.001)^∗∗∗^
Beck Depression Inventory-II (BDI-II)	–0.33	–0.394	–0.196
*M* = 8.26 (*SD* = 7.77) Range = 0–42	(*p* < 0.001)^∗∗∗^	(*p* < 0.003)^∗∗^	(*p* < 0.01)^∗∗^

*^∗∗^*p* < 0.01; ^∗∗∗^*p* < 0.001.*

### *Post hoc* Tests: Evidence for Dissociation Between Empathic Happiness and Anhedonia

When viewing Figure 1B, it becomes clear that there are a group of individuals high in empathic happiness and also high in anhedonia. Inspection of the frequency plot for the SHAPS revealed a multi-modal distribution, whereas empathic happiness was more normally distributed. The dissociation between SHAPS score and Positive empathy delivers the point that empathic happiness is at least partially dissociable from self-focused pleasure capacity. *Post hoc* analyses comparing the subgroup in Figure 1B to the rest of the sample were completed to better characterize these individuals who are anhedonic but also report feeling a lot of empathic happiness. This subset of 14 participants (who had a PES score of >45 and a SHAPS score equal to or less than 40), were derived and entered into analyses. This subgroup, relative to the remainder of the sample, had higher (1) *Empathy Quotient-Cognitive* scores [*F*(1,212) = 5.01, *p* = 0.02], and (2) empathic cheerfulness subscale scores [F(1,281) = 10, *p* = 0.002]; but did not differ in terms of BDI-II score [*F*(1,212) = 1.76, *p* = 0.186], age [*F*(1,212) = 1.57, *p* = 0.21], or gender. These findings suggest that these individuals are good at taking the perspective of, and reading the mental states of others. It is possible that this type of individual might also have better executive function (e.g., Light et al., 2017), which can be compromised in individuals who are clinically depressed. However, it is really unknown how these individuals function in their day-to-day lives, and these results raise important questions about the role of positive empathy in overall well-being. If personal pleasure capacity is lacking, but they are able to derive pleasure from seeing others happy, does this relate to a particular pattern of symptomatology? Can these individuals be trained to indulge their personal hedonic capacity system as a means to reduce risk of developing depression or some other psychopathology later?

In sum, these *post hoc* results suggest that anhedonia can be multi-modal in distribution across the general population, and can appear independent of frank depression and positive empathy capacity, and supports findings in the literature that suggest that anhedonia exists on a continuum even in the general population (Harvey et al., 2007), and dissociations are possible.

## Discussion

These results provide preliminary support for the validity of the Light-Moran PES-15 with a Cronbach’s alpha value of 0.92 and 56.23% explained variance. Furthermore, our results indicate that people who tend to exhibit *positive empathy* also tend to experience *empathic concern*, consistent with data from a child sample (Light et al., 2009).

### The Relationship Between Empathy and Anhedonia

The Light-Moran PES-15 provides a novel means to assess the functioning of the positive affect system. Particularly, the PES-15 offers researchers and clinicians a way to measure an as yet-untapped component of the positive affect system which, upon examination, could allow for better assessment and intervention of dysfunction (or skill) most relevant to subjective happiness.

Our data provide evidence that hedonic capacity/anhedonia does relate to one’s ability to vicariously experience the positive affect of another individual; and also relates to empathic concern. Based on the data collected for this study, we can say that individuals who show empathic happiness also tend to score higher on empathic concern, but these variables were not highly correlated, suggesting that there are some people who are high on one form of empathy, but not necessarily both.

Our results also indicate that positive-valence empathy deficiencies may better predict depressive symptomatology than total anhedonia. Consistent with models of depression which posit the centrality of interpersonal deficits in maintenance of the disorder (e.g., McCullough, 2000), this finding highlights the interpersonal dimension of low positive affect. This may be useful for clinicians wishing to assess this facet of low positive affect.

### Application: Positive-Valence Empathy as a Potential Treatment for Anhedonia

Although happiness is an elusive construct, most agree that it is a quality that everyone would like to increase, and certainly there are several mental disorders that feature lack of happiness or positive affect as a central feature (e.g., Major Depressive Disorder). Happiness can be defined as the frequent experience of positive emotions (Lyubomirsky et al., 2005). The repeated experience of “empathic happiness” over time may increase happiness. Overall, we believe happiness is a skill that may be harnessed/developed via the induction of empathic happiness, possibly utilizing emotion regulation techniques from the extant neuroscience literature. We believe this is a viable and meaningful paradigm because there is a growing corpus of evidence to suggest that interventions based on emotion regulation and positive psychology theory, ranging from “cognitive reappraisal” to “counting one’s blessings” to interventions derived from ancient contemplative practices (e.g., “loving-kindness meditation”) induce plasticity related alterations in the brain (e.g., Ochsner et al., 2012; Klimecki et al., 2013; Weng et al., 2013; Engen and Singer, 2015) and support a range of positive behavioral outcomes, such as immune function, prosocial behavior, and problem solving (Davidson and McEwen, 2012). Prior research has established that primary positive emotion (e.g., joy) can be up-regulated on a moment-to-moment basis utilizing reappraisal strategies borne from the extensive emotion regulation research literature (e.g., for a review of positive emotion regulation see Carl et al., 2013). For example, Heller et al. (2013) found that individuals with MDD that responded to pharmacological treatment showed an increase in ventral striatal activity – and fronto-striatal connectivity – from baseline to 8-weeks of antidepressant treatment in response to positively valenced visual stimuli; suggesting that changes in positive emotionality relate to neuroplastic changes in brain circuitry related to emotion regulation and positive affect. We extrapolate this idea by suggesting that the induction of empathic happiness, via emotion regulation strategies, may also lead to neuroplastic changes in the brain, and ultimately such brain changes may promote increases in subjective happiness.

In addition, research to date also provides preliminary evidence that qualities such as empathy and compassion, in addition to primary positive emotions such as joy, can be cultivated or otherwise increased; much like other skills are learned through sustained repetitive practice that over time leads to automatized habits (Davidson and McEwen, 2012). For example, the results of a recent study suggest that, compared with a control group, compassion training elicits activity in a neural network including the medial orbitofrontal cortex, putamen, pallidum, and ventral tegmental area – brain regions previously associated with positive affect (Klimecki et al., 2013). Thus, we argue that there is reason to believe that complex positive emotions, such as compassion and (positive) empathy, can be up-regulated just as basic positive emotions such as joy can be. Overall, we believe inducing empathic happiness via cognitive means (i.e., via routinized instruction) will ultimately increase the amount of, and/or sustenance of, positive emotion generated and experienced, and may thus have utility in addressing the symptom of anhedonia.

## Conclusion

The Light-Moran Positive Empathy Scale (PES-15) offers a novel means to measure positive-valence empathy. Importantly, our results suggest that positive affect is a heterogeneous construct and the various known forms of positive affect (e.g., joy versus *positive-valence empathy*) are likely not synonymous. Use of more than one scale to measure these various facets of positive affect is needed and may be important to implement in clinical practice so that a fuller picture of the functioning of the positive emotional system can be gleaned. Such an approach may prove useful for choosing treatment strategies that fit an individual’s unique positive affectivity profile. Essentially, ascertaining the level of functioning of one’s positive affectivity system can be helpful for determining areas of positive affect weakness, which once identified may potentially be effortfully strengthened (Heller et al., 2009; Light et al., 2011).

The two subsets of items that make up the PES-15 likely do entail different psychological processes: in one case, the observer is identifying with the negative emotion of someone else (i.e., Empathic Cheerfulness) versus identifying with the positive emotional state of someone else (i.e., Empathic Happiness). However, importantly, both psychological processes entail relating to the emotional state of someone else; and we believe this is what unifies these constructs. However, empathic cheerfulness has more of an “active” component than empathic happiness. Indeed, the results from a recent fMRI study with adults (Mirabito et al., 2019) revealed that empathic happiness relates more so to nucleus accumbens shell activity, whereas empathic cheerfulness correlated with globus pallidus activation. Together, this suggests that there are separable behavioral and neurobiological aspects to empathic cheerfulness versus empathic happiness. Therefore, depending on the level of analysis, and whether the researcher is interested in understanding more comprehensively the correlates of identifying with the emotional state of someone else regardless of valence, then the total PES-15 score may be of more interest, whereas a researcher particularly interested in the relationship between vicarious positive affect versus empathic cheerfulness (or empathic concern) may be more interested in looking at the subscale scores of the PES-15 separately. Along the same line, it should be noted that the two factor solution accounts for a moderate proportion of total variance. This suggests to us that we are indeed only measuring a *subcomponent* of hedonic capacity, and this result should be viewed as evidence that our scale is a specialized measurement of an aspect of hedonic capacity; and it does not represent hedonic capacity in its entirety. Positive-valence empathy may nevertheless represent a particularly important facet of hedonic capacity that as yet has not been extensively investigated.

Limitations of the current study include the fact that only healthy adults were utilized. Furthermore, our sample was homogeneous and had limited ethnic minority representation. Therefore, future work is needed to provide normative data for various sub-group/patient populations. Also, the sample was mostly composed of college-aged individuals, thus further investigation is warranted to determine the relationship between positive-valence empathy and anhedonia in child and elderly samples as well. Age was used as a covariate in certain analyses to control for an age difference between the large and small subsamples used in the present investigation. Importantly, the results did not change when age was included versus excluded.

Directions for future research might examine the predictive utility of positive-valence empathy. For example, longitudinal changes in empathic happiness may be hypothesized to predict changes in depression scores over the course of treatment. In conclusion, the PES-15 assesses an aspect of functioning of the positive affect system in healthy adults that is not typically addressed, and may have diagnostic value in detecting difficulties in the experience of positive emotions. Positive-valence empathy may represent a higher order positive affective state that relates to a basic ability to experience positive emotions such as joy and contentment.

## Ethics Statement

This study was carried out in accordance with the recommendations of the University of Wisconsin–Madison Institutional Review Board, with written informed consent from all subjects. All subjects gave written informed consent in accordance with the Declaration of Helsinki. The protocol was approved by the University of Wisconsin–Madison Institutional Review Board.

## Author Contributions

All authors listed have made a substantial, direct and intellectual contribution to the work, and approved it for publication.

## Conflict of Interest Statement

The authors declare that the research was conducted in the absence of any commercial or financial relationships that could be construed as a potential conflict of interest.

## Appendix A: 15-Item Positive Empathy Scale (Post-Factor Analysis)

### Positive Empathy Scale

Instructions:

• There is a list of statements below.
• Please read each statement *carefully*.
• Rate how strongly you agree or disagree with the statement.
• Check *one* of the boxes [x] to indicate your answer.
• There are no right or wrong answers, or trick questions.

1. I very much enjoy and feel uplifted by happy endings.
       [ ] Extremely true
       [ ] Quite true
       [ ] Slightly true
       [ ] Neither true nor false
       [ ] Slightly untrue
       [ ] Quite untrue
       [ ] Extremely untrue
2. I like to tell people nice things to make them feel good.
       [ ] Extremely true
       [ ] Quite true
       [ ] Slightly true
       [ ] Neither true nor false
       [ ] Slightly untrue
       [ ] Quite untrue
       [ ] Extremely untrue
3. I can’t stop myself from laughing when others are doing so.
       [ ] Extremely true
       [ ] Quite true
       [ ] Slightly true
       [ ] Neither true nor false
       [ ] Slightly untrue
       [ ] Quite untrue
       [ ] Extremely untrue
4. I feel great when I find out that I have made someone else happy.
       [ ] Extremely true
       [ ] Quite true
       [ ] Slightly true
       [ ] Neither true nor false
       [ ] Slightly untrue
       [ ] Quite untrue
       [ ] Extremely untrue
5. I also feel good when someone I know feels good.
       [ ] Extremely true
       [ ] Quite true
       [ ] Slightly true
       [ ] Neither true nor false
       [ ] Slightly untrue
       [ ] Quite untrue
       [ ] Extremely untrue
6. I enjoy hearing about my friends’ good days.
       [ ] Extremely true
       [ ] Quite true
       [ ] Slightly true
       [ ] Neither true nor false
       [ ] Slightly untrue
       [ ] Quite untrue
       [ ] Extremely untrue
7. It often makes me feel good to see the people around me smiling.
       [ ] Extremely true
       [ ] Quite true
       [ ] Slightly true
       [ ] Neither true nor false
       [ ] Slightly untrue
       [ ] Quite untrue
       [ ] Extremely untrue
8. I find that other people’s happiness easily “rubs off” on me.
       [ ] Extremely true
       [ ] Quite true
       [ ] Slightly true
       [ ] Neither true nor false
       [ ] Slightly untrue
       [ ] Quite untrue
       [ ] Extremely untrue
9. I enjoy helping people to see that they can turn “lemons into lemonade.”
       [ ] Extremely true
       [ ] Quite true
       [ ] Slightly true
       [ ] Neither true nor false
       [ ] Slightly untrue
       [ ] Quite untrue
       [ ] Extremely untrue
10. I feel good when I know I have pleased someone.
       [ ] Extremely true
       [ ] Quite true
       [ ] Slightly true
       [ ] Neither true nor false
       [ ] Slightly untrue
       [ ] Quite untrue
       [ ] Extremely untrue
11. I enjoy making others feel good.
       [ ] Extremely true
       [ ] Quite true
       [ ] Slightly true
       [ ] Neither true nor false
       [ ] Slightly untrue
       [ ] Quite untrue
       [ ] Extremely untrue
12. I enjoy helping a person change their bad mood into a good mood.
       [ ] Extremely true
       [ ] Quite true
       [ ] Slightly true
       [ ] Neither true nor false
       [ ] Slightly untrue
       [ ] Quite untrue
       [ ] Extremely untrue
13. I enjoy making others laugh.
       [ ] Extremely true
       [ ] Quite true
       [ ] Slightly true
       [ ] Neither true nor false
       [ ] Slightly untrue
       [ ] Quite untrue
       [ ] Extremely untrue
14. I easily get excited when those around me are lively and happy.
       [ ] Extremely true
       [ ] Quite true
       [ ] Slightly true
       [ ] Neither true nor false
       [ ] Slightly untrue
       [ ] Quite untrue
       [ ] Extremely untrue
15. I can’t help but smile when my friends smile at me.
       [ ] Extremely true
       [ ] Quite true
       [ ] Slightly true
       [ ] Neither true nor false
       [ ] Slightly untrue
       [ ] Quite untrue
       [ ] Extremely untrue

### Appendix B: Pes-15 Scoring Instructions

**Table:**

7	Extremely true
6	Quite true
5	Slightly true
4	Neither true nor false
3	Slightly untrue
2	Quite untrue
1	Extremely untrue

“Empathic Happiness” subscale: Sum items 1, 3, 5, 6, 7, 8, 14, and 15. Empathic happiness is a vicarious emotional response that involves happiness (or a similar positive affect) and an other-oriented feeling of goodwill toward the other person (Light et al., 2009).
“Empathic Cheerfulness” subscale: Sum items 2, 4, 9, 10, 11, 12, and 13. Empathic cheerfulness is an emotional response that involves the display of positive affect in response to someone in distress as a means to cheer the victim up, and involves a feeling of goodwill (Light et al., 2009).
Total Score: Sum all items, score range is 15–105. The mean score for healthy women is *M* = 89.63, *SD* = 9.74; and the mean score for healthy men is (*M* = 83.95, *SD* = 10.68).
